# Prevalence of neuropsychiatric disorders in internally displaced persons with dementia during wartime in Ukraine

**DOI:** 10.1192/j.eurpsy.2023.536

**Published:** 2023-07-19

**Authors:** M. Dzis, L. Rakhman

**Affiliations:** Psychiatry, Psychology and Sexology, Lviv National Medical University after Danylo Halyckyi, Lviv, Ukraine

## Abstract

**Introduction:**

During the second wave of Russia-Ukraine war, around 8 million were internally displaced. Negative mental health impact of the war cannot be underestimate. Among internally displaced persons (IDPs), particularly vulnerable category is people with neurocognitive deficits. Stress associated with displacement may cause a change not only in cognitive functions, but also affect the onset or evaluation of behavioral and psychological symptoms.

**Objectives:**

to study the prevalence of neuropsychiatric disorders in hospitalized patients with dementia, who were internally displaced and to compare with general population frequency.

**Methods:**

64 IDPs with dementia (moderate and severe neurocognitive deficits) who were examined during March-September 2022. Cases of newly arrived persons were taken into account, after 1 to 30 days had passed since their relocation. The diagnosis was verified based on the ICD-10 criteria (F00-F01). The degree of neurocognitive deficit was determined using the MMSE and MoCA tests. Affective pathology was studied using the HAM-D, HAM-A, PHQ-9, AES scales. Psychotic symptoms and behavioral disorders were studied based on clinical examination and medical records. The study was conducted in Lviv Regional Psychiatric Hospital.

**Results:**

among the examined patients, 60 (94% of all examined) had neuropsychiatric disorders. Among this sample, neuropsychiatric symptoms (an isolated symptom or a combination of two or more symptoms) occurred with the following frequency: apathy 16 (26.7%), anxiety49 (81.7%), depressive symptoms 32 (53.3%), agitation and aggression 41 (68.3%), hallucinatory symptoms 8 (13.3%), delusional disorders 34 (56.7%), wandering and disorientation 18 (30%), refusal of food and medicine 12 (20%)

**Image:**

**Figure:**
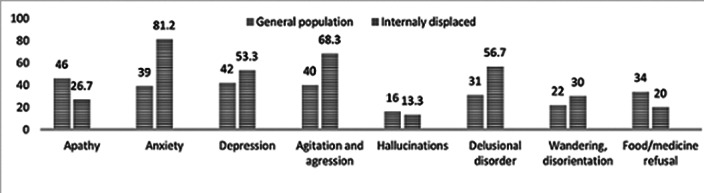

**Conclusions:**

In this study the frequency of occurrence of certain neuropsychiatric syndromes among IDPs with dementia differed from the studied average frequency of occurrence of the same symptomatology among the general population. In particular, anxiety symptoms among IDPs with dementia occurred 42% more often than on average among patients with dementia in the general population (with a frequency of 81% vs 39%), agitation and aggression - 28% more often (68% vs 40%), and delusions - 16% more often (57% vs 31%). At the same time, symptoms such as apathy (by 19%) and refusal to eat (by 14%) were observed less often among IDPs with dementia than among dementia patients from the general population

**Disclosure of Interest:**

None Declared

